# A pragmatic effectiveness-implementation study comparing trial evidence with routinely collected outcome data for patients receiving the REACH-HF home-based cardiac rehabilitation programme

**DOI:** 10.1186/s12872-022-02707-5

**Published:** 2022-06-16

**Authors:** Paulina Daw, Alexander Harrison, Patrick J. Doherty, Jet J. C. S. Veldhuijzen van Zanten, Hasnain M. Dalal, Rod S. Taylor, Samantha B. van Beurden, Sinéad T. J. McDonagh, Colin J. Greaves

**Affiliations:** 1 School of Sport, Exercise and Rehabilitation Sciences, University of Birmingham, Edgbaston, B15 2TT UK; 2 Health Sciences, University of York, York, UK; 3 University of Exeter Medical School, Royal Cornwall Hospital, Truro, UK; 4 Primary Care Research Group, University of Exeter Medical School, St Luke’s Campus, Exeter, UK; 5 MRC/CSO Social and Public Health Sciences Unit & Robertson Centre for Biostatistics, Institute of Health and Well Being, University of Glasgow, Glasgow, UK; 6 College of Medicine and Health, University of Exeter, Exeter, UK; 7 Primary Care Research Group, College of Medicine and Health, University of Exeter, St Luke’s Campus, Exeter, UK

**Keywords:** Heart failure, Cardiac rehabilitation, Treatment outcome, Quality of life, Routinely collected health data, Quantitative evaluation

## Abstract

**Background:**

Cardiac rehabilitation for heart failure continues to be greatly underused worldwide despite being a Class I recommendation in international clinical guidelines and uptake is low in women and patients with mental health comorbidities.

**Methods:**

Rehabilitation EnAblement in CHronic Heart Failure (REACH-HF) programme was implemented in four UK National Health Service early adopter sites (‘Beacon Sites’) between June 2019 and June 2020. Implementation and patient-reported outcome data were collected across sites as part of the National Audit of Cardiac Rehabilitation. The change in key outcomes before and after the supervised period of REACH-HF intervention across the Beacon Sites was assessed and compared to those of the intervention arm of the REACH-HF multicentre trial.

**Results:**

Compared to the REACH-HF multicentre trial, patients treated at the Beacon Site were more likely to be female (33.8% vs 22.9%), older (75.6 vs 70.1), had a more severe classification of heart failure (26.5% vs 17.7%), had poorer baseline health-related quality of life (MLHFQ score 36.1 vs 31.4), were more depressed (HADS score 6.4 vs 4.1) and anxious (HADS score 7.2 vs 4.7), and had lower exercise capacity (ISWT distance 190 m vs 274.7 m). There appeared to be a substantial heterogeneity in the implementation process across the four Beacon Sites as evidenced by the variation in levels of patient recruitment, operationalisation of the REACH-HF intervention and patient outcomes. Overall lower improvements in patient-reported outcomes at the Beacon Sites compared to the trial may reflect differences in the population studied (having higher morbidity at baseline) as well as the marked challenges in intervention delivery during the COVID-19 pandemic.

**Conclusion:**

The results of this study illustrate the challenges in consistently implementing an intervention (shown to be clinically effective and cost-effective in a multicentre trial) into real-world practice, especially in the midst of a global pandemic. Further research is needed to establish the real-world effectiveness of the REACH-HF intervention in different populations.

**Supplementary Information:**

The online version contains supplementary material available at 10.1186/s12872-022-02707-5.

## Contributions to the literature

- Understanding of real-world implementation and impact on patient-reported outcomes of an evidence-based intervention for heart failure.
- Rehabilitation EnAblement in CHronic Heart Failure may be a viable cardiac rehabilitation alternative for older patients and patients with mental health comorbidities and lead to an increase in the uptake of cardiac rehabilitation in these currently underserved populations.
- More effort is needed to offer the programme to a wider range of patients. This can be achieved by improving referral pathways and/or making the programme more accessible/appealing to different patients.
- The study is unique in utilising routine data to evaluate the real-world implementation of trial evidenced intervention; the findings can inform future studies employing similar methodologies.
- This study is part of a larger implementation project [1], the main output of this project is an implementation manual, which is available to healthcare teams interested in including REACH-HF in their service provision in the National Institute for Health and Care Excellence guidance [2].

## Background

### Cardiac rehabilitation for patients with heart failure

Cardiac rehabilitation for heart failure continues to be greatly underused worldwide despite being a Class I recommendation in international clinical guidelines. Less than 20% of eligible patients in Europe receive this intervention [3] and fewer still in the United States [4]. The landscape of cardiac rehabilitation provision before the COVID-19 pandemic was dominated by group-based programmes delivered in hospitals and community cardiac rehabilitation centres [5]. Providing different modes of delivery (such as telemedicine or home-based programmes) are proposed alternatives that could help to increase the uptake of cardiac rehabilitation [4, 6–9].

### Intervention – the REACH-HF programme

The Rehabilitation EnAblement in CHronic Heart Failure (REACH-HF) programme is a clinically effective and cost-effective, home-based, healthcare professional-facilitated cardiac rehabilitation programme for patients with heart failure [10]. The 12-week programme is delivered using a mixture of home visits and telephone calls. The average contact time per patient in the REACH-HF effectiveness trial was 5.3 h (made up of 4.5 h of face-to-face contact and 0.8 h of telephone facilitation) over an average of 6.5 sessions. The REACH-HF programme and the multicentre trial are described in more detail elsewhere [10–13].

### Research-to-practice gap

Moving research findings into routine clinical practice is a well-documented challenge [14, 15]. Once implemented, innovations often fail to replicate the effectiveness reported in clinical trials [16]. As many as 23 contextual factors can impact the implementation process, leading to variations in the delivery of the same programme between centres/teams [17]. For example, patients treated in real-world clinical settings might be different to those recruited into the trial, staff implementing the innovation might lack the knowledge and skills required to deliver the intervention as it was intended, or a lack of resources might hinder effective implementation [18, 19].

### Study aim

This study aimed to answer the following research questions: (1) What are the variations in the implementation process across the four Beacon Sites and how do such variations impact on patients’ outcomes?, (2) Are patients receiving the REACH-HF intervention at the Beacon Sites comparable to those recruited into the trial and more representative of the general population of heart failure?’ and (3) Are changes in outcomes of interest derived from routine data collected at the Beacon Sites for patients receiving the REACH-HF intervention comparable with the changes observed in the REACH-HF trial?.

## Methods and analysis

### Design

The current effectiveness-implementation study used a multi-centre prospective cohort design to evaluate the implementation process and compare trial evidence with routinely collected pre-treatment and post-treatment healthcare data during the real-world implementation of the intervention at four Beacon Sites.

### Beacon Sites

The Beacon Sites selection process is described in detail in the published protocol [1] and summarised in Additional file 1. Four Beacon Sites were set up to deliver REACH-HF to 200 patients between June 2019 and June 2020; characteristics and cardiac rehabilitation activity before becoming Beacon Sites are summarised in Table 1. Each site was required to administer two key outcome measures as were used in the clinical trial–the Minnesota Living with Heart Failure Questionnaire (MLHFQ) and the Incremental Shuttle Walk Test (ISWT).

**Table 1:** Beacon Sites’ characteristics and cardiac rehabilitation activity before becoming Beacon Sites (unless stated otherwise the included data are from the NACR 2016/2017 audit)

Beacon Sites’ characteristics and cardiac rehabilitation activity before becoming Beacon Sites	Site 1	Site 2	Site 3	Site 4
National certification programme for cardiac rehabilitation status	Green	Amber	Green	Green
Average core rehabilitation programme length in weeks	12	6	10	14
Number of patients attending (starting) cardiac rehabilitation	635	726	295	500
Accepting heart failure patients	Yes	No	Yes	Yes
Number of patients with heart failure who attended cardiac rehabilitation in the 12 months before the Beacon Site application	46	N/A	38	36
Offering supervised/facilitated home-based cardiac rehabilitation	Yes	Yes	Yes	No
Number of any cardiac patients who received supervised/facilitated home-based cardiac rehabilitation in the 12 months before the Beacon Site application	66	24	8	N/A
Approximate full-time equivalent staff in the multidisciplinary cardiac rehabilitation team	11	10	5	9

NACR = National audit of cardiac rehabilitation; N/A = not applicable

### Participants

Three cohorts of patients with heart failure were compared in the study: patients who received REACH-HF at the Beacon Sites, patients who received REACH-HF within the multicentre randomised controlled trial and the general population with heart failure – all heart failure patients (except those receiving REACH-HF at the Beacon Sites) in the National Audit of Cardiac Rehabilitation (NACR) database who received cardiac rehabilitation during the Beacon Site period (between June 2019 and June 2020).

### Measures

Audit data (see Results Tables) from the NACR database were used to understand the implementation process by considering how the intervention was delivered and the socio-demographic characteristics and medical data of patients treated in the Beacon Sites. The audit data were also used to compare patient characteristic and real-world changes in outcomes of patients receiving the REACH-HF programme to the prior clinical trial findings [10]. A detailed description of all the outcome measures can be found in Additional file 2.

### Data collection and analysis

A download of the NACR data was conducted by the NACR data scientist (AH) in February 2021. We focused on the four-month trial follow-up timepoint, as it was the best match for the time period between pre-treatment and post-treatment assessments at the Beacon Sites (mean 125.2 days). Due to data governance requirements, analyses using individual patient-level data were conducted by NACR staff.

Descriptive statistics were used to report the characteristics of the study sites and the patient sample. To explore whether the MLHFQ within-group change in the NACR data was different from the within-group change in the trial, a three-group (Beacon Sites vs trial treatment arm vs trial control arm) comparison (ANCOVA) was conducted with adjustment for MLHFQ baseline score, age and sex. To understand the within-group differences at the Beacon Sites and the trial, we used a standardised mean difference approach and calculated effect sizes for primary and secondary outcome measures adjusted for the sample size (Hedges’ *g*) [20, 21].

## Results

### Implementation process and sample characteristics

Implementation activity at the Beacon Sites is summarised in Table 2 (data for the individual sites can be found in Table 6 in Additional file 3). Based on the data recorded by the cardiac rehabilitation providers, of the 132 patients enrolled on the REACH-HF programme between June 2019 and June 2020, 96 (72.7%) completed the programme. The completion rate was calculated based on the patient having two valid assessments (baseline and end-of-treatment) or a ‘completed’ box being ticked for patients who were unable to attend their assessment appointments.

**Table 2:** REACH-HF Beacon Sites activity between June 2019 and June 2020

Started treatment n	132
Completed treatment n (%)	96 (72.7)
Average sessions received mean (SD)	7.5 (3.9)
MIN/MAX sessions received n	1 (MIN), 24 (MAX)
Average treatment duration in days mean (SD)	125.3 (47.4)
MIN/MAX treatment duration in days n	70 (MIN), 299 (MAX)
Dropped out n (%)	36 (27.3)
Known reason for not completing n (%)	14 (38.9)
Did not attend – unknown reason n (%)	6 (21.4)
Left the area n (%)	1 (3.6)
Planned/emergency intervention n (%)	1 (3.6)
Too ill n (%)	7 (25)
Died n (%)	3 (10.7)
Hospital readmission n (%)	2 (7.1)
Other n (%)	8 (28.6)
Missing n (%)	8 (22.2)

SD = standard deviation; MIN = minimum; MAX = maximum

On average, patients received 7.5 (SD 3.9) clinical sessions (a combination of centre-based assessments, home visits and telephone support) over 125.3 days (SD 47.4). There was a large variation in the number of sessions attended with a minimum of one and a maximum of 24 sessions. A similar variation was observed with treatment duration, 70 days was the minimum and 299 days the maximum.

The comparison of key participant characteristics across the three cohorts can be found in Table 3. Of the 132 patients enrolled on the programme, 86 (66.2%) were male and 44 (33.8%) were female. The mean age of patients treated at the four Beacon Sites was 75.6 (SD 11.1) years old; Beacon Site 1 patients were a mean of 10.5 years older than the remaining sites. Table 7 in Additional file 4 includes socio-demographic characteristics of patients enrolled on the REACH-HF programme at individual Beacon Sites and missing data, as well as more detailed comparison data.

**Table 3:** Comparison of patients receiving the REACH-HF intervention at the Beacon Sites between June 2019 and June 2020, those recruited into the REACH-HF trial and the general heart failure population recorded in the NACR database between June 2019 and June 2020 (excluding Beacon Sites patients)

	Beacon sites (n = 132, unless specified otherwise)	Trial data-treatment arm (n = 96**, unless specified otherwise)	Heart failure patients recorded in the NACR for the same period (excluding Beacon Site patients) (n = 5549)
Age in years – mean (SD)	75.6 (11.1)	70.1 (10.4)	68.8 (12.7)
Male sex—n (%*)	86 (66.2)	74 (77.1)	3860 (69.6)
Ethnic group—n (%)	White 81 (90)	White 91 (94.8)	White 3935 (83.5)
	Non-white 9 (10)	Other/Black/Asian ethnic group 5 (5.2)	BAME 780 (16.5)
Accommodation status/marital status—n (%)	Single 29 (35.8)	Living alone 25 (26.1)	Single 1081 (28.9)
	Married/living with a partner 52 (64.2)	Living with a partner and/or a family member 71 (73.9)	Married/living with a partner 2664 (71.1)
Employment status—n (%)	Employed 5 (7.8)	Employed 15 (15.6)	Employed 546 (16.3)
	Unemployed/retired 59 (92.2)	Unemployed/retired 81 (84.4)	Unemployed/retired 2798 (83.7)
Mean MLHFQ at baseline (SD, n)	36.1 (22.7, 50)	31.4 (22.8, 96)	
Mean HADS (depression) at baseline (SD, n)	6.4 (5, 23)	4.1 (3.3, 96)	5.5 (4, 2761)
Mean HADS (anxiety) at baseline (SD, n)	7.2 (5.2, 23)	4.7 (4.1, 96)	5.9 (4.5, 2762)
Mean ISWT at baseline (SD, n)	190 (119.4, 13)	274.7 (153.7, 90)	
Heart failure status (NYHA) – n (%)			
Class I	2 (5.9)	22 (22.9)	217 (22.3)
Class II	23 (67.6)	57 (59.4)	494 (50.7)
Class III	9 (26.5)	17 (17.7)	211 (21.6)
Class IV	–	–	53 (5.4)

NACR = National Audit of Cardiac Rehabilitation; SD = standard deviation; BAME = Black, Asian and minority ethnic; MLHFQ = Minnesota Living with Heart Failure Questionnaire; HADS = Hospital Anxiety and Depression Scale; ISWT = Incremental Shuttle Walk Test; NYHA = New York Heart Association Heart Failure Classification

*Valid percent values; **Baseline characteristics for 96 patients who had MLHFQ recorded at baseline and four-month follow-up, unless specified otherwise

Referral sources for patients treated at the Beacon Sites can be found in Table 4 (data for the individual sites are in Table 8 in Additional file 5).

**Table 4:** Referral sources for patients enrolled on the REACH-HF programme at the Beacon Sites between June 2019 and June 2020

Total (n)		n = 105
Source of referral n (%)	Consultant	5 (4.8)
	Cardiac nurse	96 (91.4)
	GP	1 (1)
	Primary care nurse	3 (2.9)
	Missing	27 (20.5)

### Patient outcomes

Table 5 compares primary and secondary outcomes achieved at the Beacon Sites with those from the trial. The ANCOVA analysis confirmed that the trial population did significantly better (Mean Difference − 7.2: 95%CI − 14.1 to − 0.3) compared with the Beacon Site population in terms of improvement in health-related quality of life measured by the MLHFQ. Furthermore, the ANCOVA comparison (NACR data vs trial control group) confirmed that there was no significant difference in the improvement of the MLHFQ scores at the Beacon Sites (Mean Difference 1.9: 95%CI -5 to 8.9).

**Table 5:** Comparison of the primary and secondary outcomes for patients who received the REACH-HF intervention at the Beacon Sites between June 2019 and June 2020 and those recruited into the REACH-HF trial

Measure	Pre			Post			Effect size Hedges’ *g* (95% CI)
	N	Mean	SD	N	Mean	SD	
**MLHFQ**							
Beacon Sites	50	36.1	22.7	50	34.0	21.7	− 0.09 (− 0.49 to 0.30)
Trial	96	31.4	22.8	96	22.7	18.4	− 0.42 (− 0.70 to − 0.13)
**HADS (depression)**							
Beacon Sites	23	6.4	5.0	23	5.7	4.2	− 0.15 (− 0.73 to 0.43)
Trial	95	4.2	3.3	95	3.6	2.7	− 0.20 (− 0.48 to 0.09)
**HADS (anxiety)**							
Beacon Sites	23	7.2	5.2	23	6.5	4.4	− 0.14 (− 0.72 to 0.44)
Trial	95	4.7	4.2	95	4.4	3.9	− 0.07 (− 0.36 to 0.21)
**ISWT (m)**							
Beacon Sites	13	190.0	119.4	13	211.5	122.9	0.17 (− 0.60 to 0.94)
Trial	84	277.8	152.5	84	322.3	173.2	0.27 (− 0.03 to 0.58)

SD = standard deviation; CI = confidence intervals; MLHFQ = Minnesota Living with Heart Failure Questionnaire; HADS = Hospital Anxiety and Depression Scale; ISWT = Incremental Shuttle Walk Test

The pre-treatment and post-treatment effect sizes calculated at the Beacon Sites (Table 5) did not match the effect sizes calculated from the trial data for the health-related quality of life – MLHFQ (Mean Difference − 0.09: 95% CI − 0.49 to 0.30 vs − 0.42: 95% CI − 0.70 to − 0.13), mental health measures (Hospital Anxiety and Depression Scale (HADS) depression – Mean Difference − 0.15: 95% CI − 0.73 to 0.43 vs − 0.20: 95% CI − 0.48 to 0.09) and exercise capacity measure – ISWT (Mean Difference 0.17: 95% CI − 0.60 to 0.94 vs 0.27: 95% CI − 0.03 to 0.58). The pre-treatment and post-treatment effect size for HADS anxiety score recorded at the Beacon Sites exceeded the effect size calculated from the trial data (Mean Difference − 0.14: 95% CI − 0.72 to 0.44 vs − 0.07: 95% CI − 0.36 to 0.21).

We observed the following pattern of variation in the magnitude of effect sizes at the individual Beacon Sites – a deterioration in all outcome measures at Site 1, no change in the MLHFQ and a small positive change in the mental health measures at Site 2, a small positive change in all available outcome measures at Site 3 and a small, a medium and a large effect size at Site 4 for the MLHFQ, and the HADS depression and anxiety domains, respectively. Table 9 in Additional file 6 lists effect sizes for primary and secondary outcome measures at individual Beacon Sites.

## Discussion

The primary and secondary analysis confirmed that the results found in the REACH-HF trial intervention group were not replicated in the Beacon Site population. However, it was difficult to conclude definitively that the real-world implementation resulted in lower effectiveness, as there were substantial differences between the trial and the Beacon Sites in terms of the patient population and the level of implementation (treatment dose/duration). We also encountered a small sample size and missing data, which introduced selection bias into the study, further complicated by a lack of a non-treatment control group. The discrepancies in the implementation process and population characteristics observed in the study are consistent with other studies looking at the implementation of interventions that have been found to be effective in trials[22]. At the Beacon Sites, the usual implementation challenges were exacerbated by severe service disruptions due to the COVID-19 pandemic.

### Possible explanations of the data

Firstly, the COVID-19 pandemic led to substantial variations in the delivery of the programme across the Beacon Sites. Our findings suggest that when delivered as part of routine practice, the programme was spread out over a longer time period (an additional 41 days); there was also a large variation in the number of treatment sessions received. This might suggest reduced treatment fidelity, leading to lower effectiveness. The inability to deliver the treatment as it was intended (i.e., face-to-face and in the patient’s home) might have resulted in a diminished quality of care compared to the trial. For example, having to substitute face-to-face home visits for phone sessions might have impacted the nurse’s ability to deliver the person-centred interactions that are at the core of the intervention [13].

Secondly, compared to the trial population, patients treated at the Beacon Sites were older, more depressed and anxious, had lower exercise capacity, and experienced more debilitating symptoms of heart failure. The 4.7-point difference in mean MLHFQ between cohorts is close to the minimal clinically important difference of five points [23], which suggests that the health-related quality of life of patients treated at the Beacon Sites was lower than that of those recruited into the trial. In more clinically morbid populations with heart failure, the normal prognosis is for a worsening of the condition and an associated decline in quality of life over time [24]. Hence, in this population, a pattern of ‘no decline’ in symptoms over time may be a positive outcome. As we do not have data from a control group with the same baseline characteristics, we cannot be sure that this is the case here. However, the pattern of data between sites indicates that this is a plausible explanation. At Site 1, which treated the oldest/the most frail patients in our cohort, there was a consistent deterioration in all outcome measures (i.e., MLHFQ, Dartmouth Cooperative Functional Assessment Charts, HADS and ISWT), whereas, across the other sites the trend was more positive (see Table 9 in Additional file 6).

Historically, the uptake of cardiac rehabilitation in older patients with heart failure and in patients with mental health issues has been particularly low [25, 26]. As patients who received REACH-HF at the Beacon Sites compared to those recorded in the wider NACR dataset for the same period were older (on average 6.8 years) and had more mental health morbidity (HADS depression score 6.4 vs 5.5 and HADS anxiety score 7.2 vs 5.9), it may be suggested that the REACH-HF home-based cardiac rehabilitation programme might be a more acceptable form of rehabilitation to older/more frail patients and patients with mental health comorbidities compared with centre-based programmes (or at least that cardiac rehabilitation staff feel more comfortable recommending this rehabilitation option to these patients). In the real-word, only a limited range of patients accessed the programme, hence, the clinical implication of the study, irrespective of the cause of this pattern (patient preference or referral procedures), is a need to make the intervention accessible to a wider range of participants.

### Strengths and limitations

Using routinely collected audit data was a low-cost and a low research-burden option that allowed us to conduct a rapid review of the implementation process and to compare REACH-HF trial outcomes with real-world implementation outcomes. The study is of high clinical relevance, as the initial results suggest that the REACH-HF programme may be a viable cardiac rehabilitation alternative for older patients and patients with mental health comorbidities, both often underrepresented in traditional centre-based programmes (or that healthcare professionals are more open to offering REACH-HF to such patients). However, steps need to be taken to make the programme more accessible to a wide range of patients. The study also provides good insight into conducting implementation research in challenging contexts and during unprecedented (for healthcare services, healthcare professionals and the general population) times.

The main limitations of the study were the substantial variations in implementation of the treatment, high levels of missing data, a limited sample size that may have introduced selection bias (all impacted by the COVID-19 pandemic), and a lack of a comparable patient group (the trial population we had selected for comparison was substantially different to the implementation sample).

### Future research

More research, as is currently being conducted in the SCOT REACH-HF project, which is examining the real-world roll out of REACH-HF across six health boards in Scotland [27], is needed to understand the impact of REACH-HF (and other home-based rehabilitation treatments) on different populations and in relation to delivery characteristics (quantity and quality) and outside the context of global pandemic and lockdowns.

Additionally, as the amount of NACR routinely collected data from cardiac rehabilitation teams offering the REACH-HF intervention UK-wide increases, more rigorous analysis of implementation effectiveness can be applied (i.e., patient-level analysis, further consideration of different implementation contexts, correlation between the intervention (e.g., number of clinical sessions attended) and outcomes). However, steps should be taken to reduce the amount of missing follow-up data, particularly for key outcome measures such as health-related quality of life.

## Conclusions

Our data suggest that changes in patient-related outcomes seen in the REACH-HF randomised trial were not replicated in the real-world setting of this study. They also suggest that offering home-based cardiac rehabilitation may facilitate uptake amongst older patients and those with mental health comorbidities. However, our results need to be interpreted carefully given substantial differences in the populations treated and the context of the COVID-19 pandemic, which may have affected the intensity of treatment delivery.

## Supplementary Information

**Additional file 1:** Setting up the Beacon Sites.

**Additional file 2:** Detailed description of primary and secondary outcome measures.

**Additional file 3: Table 6.** REACH-HF activity at individual Beacon Sites between June 2019 and June 2020.

**Additional file 4: Table 7a.** Socio-demographic characteristics of patients enrolled onto the REACH-HF programme at individual Beacon Sites between June 2019 and June 2020 (including missing data) AND **Table 7b.** Comparison of patients receiving the REACH-HF intervention at the Beacon Sites between June 2019 and June 2020, those recruited into the REACH-HF trial and the general heart failure population recorded in the NACR database between June 2019 and June 2020 (excluding Beacon Sites patients).

**Additional file 5: Table 8.** Referral sources for patients enrolled on the REACH-HF programme at individual Beacon Sites between June 2019 and June 2020.

**Additional file 6: Table 9.** Effect sizes for primary and secondary outcome measures at individual Beacon Sites for patients receiving REACH-HF programme between June 2019 and June 2020.

## Data Availability

The datasets analysed during the current study are available from the corresponding author on reasonable request.

## Abbreviations

PD: Paulina Daw
AH: Alexander Harrison
PJD: Patrick J Doherty
JVvZ: Jet JCS Veldhuijzen van Zanten
HMD: Hasnain M Dalal
RST: Rod S Taylor
SBvB: Samantha B van Beurden
STJMcD: Sinéad T J McDonagh
CG: Colin Greaves
REACH-HF: Rehabilitation EnAblement in CHronic Heart Failure
NACR: National Audit of Cardiac Rehabilitation
MLHFQ: Minnesota Living with Heart Failure Questionnaire
HADS: Hospital Anxiety and Depression Scale
ISWT: Incremental Shuttle Walk Test
ANCOVA: Analysis of covariance
