# Supplementary material for: A pragmatic effectiveness-implementation study comparing trial evidence with routinely collected outcome data for patients receiving the REACH-HF home-based cardiac rehabilitation programme
Source: BMC Cardiovasc Disord. 2022 Jun 16;22:270. doi: 10.1186/s12872-022-02707-5 (PMC9202968; doi:10.1186/s12872-022-02707-5)
Supplement: Supplementary file 1 — Additional file 1: Setting up the Beacon Sites. [file 12872_2022_2707_MOESM1_ESM.docx]

**Additional file 1 - Setting up the Beacon Sites**

To become a Beacon Site, a cardiac rehabilitation team had to:

- be registered with, and reporting high-quality audit data to the NACR (being awarded green or amber certification, through meeting respectively four or seven out of seven Key Performance Indicators as set by the British Association for Cardiovascular Prevention and Rehabilitation and the NACR)(34);
- allow three members of the team to attend a three-day in-person facilitated REACH-HF practitioner course, with an additional day of directed (asynchronous) learning;
- be committed to delivering the REACH-HF programme to 50 patients and their caregivers during a 12-month period following the initial training;
- be able to use the same outcome measures at baseline and post-treatment as the ones used in the REACH-HF clinical trial(23);
- be committed to participating in research activities evaluating the process of implementation (gathering patient data, qualitative interviews with staff, audio recording of clinical interactions) and sharing their knowledge/experiences of implementation with the relevant interested parties.

To incentivise participation in the Beacon Site project, sites did not have to pay for the REACH-HF training (including training manuals) for three health professionals to deliver REACH-HF, post-training support as well as intervention materials for the treatment of 50 patients (this included the REACH-HF patient manual, the Family and Friends Resource, audio with relaxation techniques and chair-based exercise DVD/video). The REACH-HF training and treatment materials were externally funded through research funds.

The cardiac rehabilitation staff who delivered REACH-HF at the Beacon Sites received the same training as the facilitators who delivered REACH-HF in the clinical trial, i.e., a 3-day face-to-face training course facilitated by the Heart Manual Department, NHS Lothian in Edinburgh. The training included sessions on psychology, behaviour change, physical activity and exercise, engaging the caregiver, and how to successfully facilitate the delivery of the intervention. Following the initial training, the NHS staff working at the Beacon Sites were able to contact the research team, the REACH-HF trainers, and the NACR team with any implementation and data-entry related questions. REACH-HF practitioners from three Beacon Sites were available to take part in an hour and a half long peer-support session facilitated by the REACH-HF trainers in December 2019. The purpose of this virtual meeting was to help embed the learning from the initial training and troubleshoot any implementation problems.
