# Supplementary material for: A pragmatic effectiveness-implementation study comparing trial evidence with routinely collected outcome data for patients receiving the REACH-HF home-based cardiac rehabilitation programme
Source: BMC Cardiovasc Disord. 2022 Jun 16;22:270. doi: 10.1186/s12872-022-02707-5 (PMC9202968; doi:10.1186/s12872-022-02707-5)
Supplement: Supplementary file 2 — Additional file 2: Detailed description of primary and secondary outcome measures. [file 12872_2022_2707_MOESM2_ESM.docx]

# **Additional file 2 - Detailed description of primary and secondary outcome measures**

### *NACR data*

To capture the context of the Beacon Sites and the process of implementation, the following data were included: source of referral, number of patients enrolled on the programme, number of patients completing the programme, number of patients dropping out from the programme, reasons for not completing the programme, average programme duration in days and average number of clinical sessions received.

The socio-demographic and medical data of patients who received REACH-HF included: mean age in years, gender, ethnicity, marital status, employment status, rural/urban classification(1), index of multiple deprivation classification (quintile)(2), the severity of heart failure measured with the New York Heart Association Heart Failure Classification (NYHA) at baseline(3), and baseline recordings of mean diastolic and systolic blood pressure, drugs and comorbidities. An additional ‘missing’ category was created for measures when data were either not stated, not known, not available or not routinely recorded.

Pre-treatment and post-treatment measures for health-related quality of life, mental health status and exercise capacity were requested from the NACR database, as follows: health-related quality of life ((MLHFQ)(4), Dartmouth Cooperative Functional Assessment Charts (COOP)(5)), mental health status ((Patient Health Questionaire-9 (PHQ-9)(6), Generalised Anxiety Disorder Assessment-7 (GAD-7)(7), Hospital Anxiety and Depression Scale (HADS)(8)), and objective exercise capacity ((ISWT)(9) and Six Minute Walk Test (6MWT)(10)). For outcome measures assessing the health-related quality of life and mental health status, higher scores represent higher levels of impairment. The opposite is true for the exercise capacity measures, where higher scores represent higher physical fitness/functional capacity. Our primary outcome measure of interest was the MLHFQ. Beyond that, all the other outcomes were secondary and all analysis performed was considered to be exploratory.

### *Primary outcome measures – health-related quality of life*

The Minnesota Living with Heart Failure Questionnaire (MLHFQ)(4) is a 21-item self-administered questionnaire that measures the patient’s perception of the impact of heart failure on their quality of life. This widely used questionnaire uses a six-point rating scale (from ‘no’ and ‘very little’ to ‘very much’) to assess the impact of heart failure on four dimensions of a patient’s life: physical symptoms of heart failure, social functioning, psychosocial and cognitive functioning, and overall adverse impact of heart failure(11). The maximum score of 105 points indicates a poor health-related quality of life. The MLHFQ achieved good reliability and validity in studies testing its psychometric properties(12-17).

The Dartmouth Cooperative Functional Assessment Charts (COOP)(5) were developed to measure patient functional status in different clinical populations(18). This simple and easy to administer measure(19) can be used to assess health-related quality of life(20) and is made of nine pictorial charts representing the following domains: physical function, emotional function, daily activities, social activities, social support, change in health, overall health, pain, and quality of life(21). The functional status in each domain is rated on an illustrated (and numbered) five-point Likert scale, where a score of one indicates no impairment and a score of five indicates the most impairment. The COOP charts show good reliability and validity(18, 19, 22, 23), they are used to measure functional status in different chronic diseases and settings(24) and they are used in research(25-29). In the current study, we considered the total COOP score minus two domains (i.e., the social support and change in health) due to known issues regarding the validity – the two domains are often misinterpreted by patients(30).

### *Secondary outcome measures*

#### Mental health status

The Patient Health Questionaire-9 (PHQ-9)(6) is a nine-item self-administered questionnaire widely used in primary care settings and research(31) to diagnose and rate the severity of depression(6). Each PHQ-9 item represents a symptom of depressive disorder and is rated in terms of frequency of occurrence (not at all, several days, more than half the days and nearly every day). A total score between 20 and 27 is indicative of a severe depressive disorder, 15 to 19 – moderately severe, 10 to 14 – moderate, 5 to 9 – mild and 0 to 4 – non-clinical. The PHQ-9 is a reliable and valid measure for screening for depression(32) and for assessing depression severity(33).

The Generalised Anxiety Disorder Assessment-7 (GAD-7)(7) is a clinical measure for assessing generalised anxiety disorder and, similarly to the PHQ-9, is extensively used in primary care settings(34). This brief (seven items) self-administered questionnaire shows good validity for screening for generalised anxiety disorder and assessing its severity(7). The maximum score for the GAD-7 is 21; a score between 21 and 15 is interpreted as a severe generalised anxiety disorder, 10 to 14 – moderate, 5 to 9 – mild and 0 to 4 – subclinical(35). The PHQ-9 and the GAD-7 are used to monitor psychological therapy treatment and recovery and are the main outcome measures used in the Improving Access to Psychological Therapy services in England(36).

The Hospital Anxiety and Depression Scale (HADS) is a 14-item self-administered scale that measures anxiety and depression(8) and is widely used in clinical practice and research(37). Each HADS questionnaire consists of two lists of seven symptoms for each disorder; each symptom statement is measured on a four-point scale (zero to three). A total depression score between 21 and 11 indicates a clinical level of depression, a score of between 10 and 8 – borderline level and 7 and 0 – non-clinical. The same cut-off points are used when interpreting a total anxiety score. The validity and psychometric properties of the measure have been confirmed in numerous studies over the years(37-39). Authors of a recent systematic review concluded that the measure might be more suitable for detecting and measuring ‘emotional distress’, due to issues with its latent structure (ability to distinguish between anxiety and depression)(40).

#### Objective exercise capacity measures

Objective exercise capacity assessment is routinely used to assesses patients with heart failure and evaluate heart failure treatment(41) and there are different exercise testing protocols used with this clinical population(42). Two frequently used protocols for testing exercise capacity in patients with heart failure are the Incremental Shuttle Walk Test (ISWT)(9) and the 6 Minute Walk Test (6MWT)(10).

The main difference between the ISWT and the 6MWT is that the former is externally paced and the intensity of exercise is incrementally increased during the test, additionally, assessors do not give any verbal encouragement to the patient undergoing assessment. Whereas the 6MWT allows the patient to walk at their own pace, assessors can offer standard statements of encouragement and the objective of the test is for the patient to cover as much distance as possible during the six-minute window, as opposed to keeping up with the increasing pace in the ISWT.

The validity and reliability of the 6MWT in cardiac rehabilitation patients were confirmed by Hamilton and colleagues(43). A 2012 meta-analysis further highlighted that the 6MWT is responsive to clinical change following cardiac rehabilitation, but its intra-tester and inter-tester reliability and validity requires further research(44). The ISWT shows good test-retest reliability(45) and can provide a valid estimate for physical fitness and functional capacity for patients attending cardiac rehabilitation(46).

### *Data from the REACH-HF trial*

107 participants were randomised into the intervention arm in the REACH-HF clinical trial(47). The available trial outcome measures included MLHFQ, HADS and ISWT. We excluded 11/107 (10.3%) trial patients as they were missing the four-month follow-up data.

1. Department for Environment Food & Rural Affairs. Official Statistics: 2011 Rural Urban Classification. 2013.

2. Ministry of Housing Communities & Local Government. National Statistics: English indices of deprivation 2019. 2019.

3. The Criteria Committee of the New York Heart Association. Nomenclature and criteria for diagnosis of diseases of the heart and blood vessels. Boston: Little Brown; 1964.

4. Rector TS, Kubo SH, Cohn JN. Patients' Self-Assessment of Their Congestive Heart Failure: Content, Reliability and Validity of a New Measure, the Minnesota Living With Heart Failure Questionnaire. Heart Failure. 1987;3:198-209.

5. Nelson EC, Wasson JH, Johnson DJ, RD. H. Dartmouth COOP Functional Health Assessment Charts: Brief measures for clinical practice. Published In: Quality of Life and Pharmacoeconomics In Clinical Trials. Second Edition. Chapter 19, p.161-168. Bert Spilker, editor. Philadelphia, PA: Lippincott-Raven Publisher; 1995.

6. Kroenke K, Spitzer RL. The PHQ-9: A New Depression Diagnostic and Severity Measure. Psychiatric annals. 2002;32(9):509-15.

7. Spitzer RL, Kroenke K, Williams JBW, Löwe B. A Brief Measure for Assessing Generalized Anxiety Disorder: The GAD-7. Archives of Internal Medicine. 2006;166(10):1092-7.

8. Zigmond AS, Snaith RP. The hospital anxiety and depression scale. Acta Psychiatr Scand. 1983;67(6):361-70.

9. Singh SJ, Morgan MD, Scott S, Walters D, Hardman AE. Development of a shuttle walking test of disability in patients with chronic airways obstruction. Thorax. 1992;47(12):1019.

10. Lipkin DP, Scriven AJ, Crake T, Poole-Wilson PA. Six minute walking test for assessing exercise capacity in chronic heart failure. British Medical Journal (Clinical research ed). 1986;292(6521):653.

11. Garin O, Herdman M, Vilagut G, Ferrer M, Ribera A, Rajmil L, et al. Assessing health-related quality of life in patients with heart failure: a systematic, standardized comparison of available measures. Heart Fail Rev. 2014;19(3):359-67.

12. Garin O, Ferrer M, Pont À, Wiklund I, Van Ganse E, Vilagut G, et al. Evidence on the global measurement model of the Minnesota Living with Heart Failure Questionnaire. Qual Life Res. 2013;22(10):2675-84.

13. Munyombwe T, Höfer S, Fitzsimons D, Thompson DR, Lane D, Smith K, et al. An evaluation of the Minnesota Living with Heart Failure Questionnaire using Rasch analysis. Qual Life Res. 2014;23(6):1753-65.

14. Heo S, Moser DK, Riegel B, Hall LA, Christman N. Testing the psychometric properties of the Minnesota Living with Heart Failure questionnaire. Nurs Res. 2005;54(4):265-72.

15. Bennett SJ, Oldridge NB, Eckert GJ, Embree JL, Browning S, Hou N, et al. Comparison of quality of life measures in heart failure. Nurs Res. 2003;52(4):207-16.

16. Naveiro-Rilo JC, Diez-Juárez DM, Romero Blanco A, Rebollo-Gutiérrez F, Rodríguez-Martínez A, Rodríguez-García MA. Validation of the Minnesota living with heart failure questionnaire in primary care. Rev Esp Cardiol. 2010;63(12):1419-27.

17. Rector ST, Cohn JN. Assessment of patient outcome with the Minnesota Living with Heart Failure questionnaire: reliability and validity during a randomized, double-blind, placebo-controlled trial of pimobendan. American Heart Journal. 1992;124:1017-25

18. Nelson EC, Landgraf JM, Hays RD, Wasson JH, Kirk JW. The functional status of patients. How can it be measured in physicians' offices? Med Care. 1990;28(12):1111-26.

19. Eaton T, Young P, Fergusson W, Garrett JE, Kolbe J. The Dartmouth COOP Charts: A simple, reliable, valid and responsive quality of life tool for chronic obstructive pulmonary disease. Quality of Life Research. 2005;14(3):575-85.

20. Frost MH, Bonomi AE, Cappelleri JC, Schünemann HJ, Moynihan TJ, Aaronson NK. Applying Quality-of-Life Data Formally and Systematically Into Clinical Practice. Mayo Clinic Proceedings. 2007;82(10):1214-28.

21. Sneeuw KCA, Aaronson NK, Sprangers MAG, Detmar SB, Wever LDV, Schornagel JH. Evaluating the quality of life of cancer patients: assessments by patients, significant others, physicians and nurses. British Journal of Cancer. 1999;81(1):87-94.

22. Nelson E, Wasson J, Kirk J, Keller A, Clark D, Dietrich A, et al. Assessment of function in routine clinical practice: description of the COOP Chart method and preliminary findings. J Chronic Dis. 1987;40 Suppl 1:55s-69s.

23. Jenkinson C, Mayou R, Day A, Garratt A, Juszczak E. Evaluation of the Dartmouth COOP Charts in a large‐scale community survey in the United Kingdom. Journal of Public Health. 2002;24(2):106-11.

24. Van Weel C. Functional status in primary care: COOP/WONCA charts. Disability and Rehabilitation. 1993;15(2):96-101.

25. Meyboom-De Jong B., R.J.A. S. Studies with the Dartmouth COOP Charts in General Practice: Comparison with the Nottingham Health Profile and the General Health Questionnaire. In: Functional Status Measurement in Primary Care. Frontiers of Primary Care. New York, NY: Springer; 1990.

26. Gilliland SS, Willmer AJ, McCalman R, Davis SM, Hickey ME, Perez GE, et al. Adaptation of the Dartmouth COOP Charts for use among American Indian people with diabetes. Diabetes Care. 1998;21(5):770-6.

27. Kempen GI, van Sonderen E, Sanderman R. Measuring health status with the Dartmouth COOP charts in low-functioning elderly. Do the illustrations affect the outcomes? Qual Life Res. 1997;6(4):323-8.

28. Westbury RC, Rogers TB, Briggs TE, Allison DJ, Gérvas J, Shigemoto H, et al. A multinational study of the factorial structure and other characteristics of the Dartmouth COOP Functional Health Assessment Charts/WONCA. Fam Pract. 1997;14(6):478-85.

29. Bentsen BG, Natvig B, Winnem M. Questions you didn’t ask? COOP/WONCA Charts in clinical work and research. Family Practice. 1999;16:190-5.

30. British Heart Foundation. The National Audit of Cardiac Rehabilitation: Quality and Outcomes Report 2019. London: BHF; 2019.

31. Gilbody S, Richards D, Brealey S, Hewitt C. Screening for Depression in Medical Settings with the Patient Health Questionnaire (PHQ): A Diagnostic Meta-Analysis. J Gen Intern Med. 2007;22(11):1596-602.

32. Molebatsi K, Motlhatlhedi K, Wambua GN. The validity and reliability of the Patient Health Questionnaire-9 for screening depression in primary health care patients in Botswana. BMC Psychiatry. 2020;20(1):295.

33. Kroenke K, Spitzer RL, Williams JB. The PHQ-9: validity of a brief depression severity measure. Journal of general internal medicine. 2001;16(9):606-13.

34. Kroenke K, Spitzer RL, Williams JBW, Monahan PO, Löwe B. Anxiety Disorders in Primary Care: Prevalence, Impairment, Comorbidity, and Detection. Annals of Internal Medicine. 2007;146(5):317-25.

35. Plummer F, Manea L, Trepel D, McMillan D. Screening for anxiety disorders with the GAD-7 and GAD-2: a systematic review and diagnostic metaanalysis. General Hospital Psychiatry. 2016;39:24-31.

36. National Collaborating Centre for Mental Health. The Improving Access to Psychological Therapies Manual 2020 [Available from: <https://www.england.nhs.uk/wp-content/uploads/2020/05/iapt-manual-v4.pdf>.

37. Herrmann C. International experiences with the Hospital Anxiety and Depression Scale--a review of validation data and clinical results. J Psychosom Res. 1997;42(1):17-41.

38. Bjelland I, Dahl AA, Haug TT, Neckelmann D. The validity of the Hospital Anxiety and Depression Scale: An updated literature review. Journal of Psychosomatic Research. 2002;52(2):69-77.

39. Brennan C, Worrall-Davies A, McMillan D, Gilbody S, House A. The Hospital Anxiety and Depression Scale: A diagnostic meta-analysis of case-finding ability. Journal of Psychosomatic Research. 2010;69(4):371-8.

40. Cosco TD, Doyle F, Ward M, McGee H. Latent structure of the Hospital Anxiety And Depression Scale: A 10-year systematic review. Journal of Psychosomatic Research. 2012;72(3):180-4.

41. Wright DJ, Tan LB. The role of exercise testing in the evaluation and management of heart failure. Postgraduate Medical Journal. 1999;75(886):453.

42. Mentzer GG, Auseon AJ. Exercise Capacity in Chronic Heart Failure. US Cardiology Review. 2012;9(1):57-60.

43. Hamilton DM, Haennel RG. Validity and Reliability of the 6-Minute Walk Test in a Cardiac Rehabilitation Population. Journal of Cardiopulmonary Rehabilitation and Prevention. 2000;20(3).

44. Bellet RN, Adams L, Morris NR. The 6-minute walk test in outpatient cardiac rehabilitation: validity, reliability and responsiveness—a systematic review. Physiotherapy. 2012;98(4):277-86.

45. Hanson LC, Taylor NF, McBurney H. The 10m incremental shuttle walk test is a highly reliable field exercise test for patients referred to cardiac rehabilitation: a retest reliability study. Physiotherapy. 2016;102(3):243-8.

46. Hanson LC, McBurney H, Taylor NF. Is the 10 m incremental shuttle walk test a useful test of exercise capacity for patients referred to cardiac rehabilitation? European Journal of Cardiovascular Nursing. 2018;17(2):159-69.

47. Dalal HM, Taylor RS, Jolly K, Davis RC, Doherty P, Miles J, et al. The effects and costs of home-based rehabilitation for heart failure with reduced ejection fraction: The REACH-HF multicentre randomized controlled trial. Eur J Prev Cardiol. 2019;26(3):262-72.
