# Supplementary material for: A pragmatic effectiveness-implementation study comparing trial evidence with routinely collected outcome data for patients receiving the REACH-HF home-based cardiac rehabilitation programme
Source: BMC Cardiovasc Disord. 2022 Jun 16;22:270. doi: 10.1186/s12872-022-02707-5 (PMC9202968; doi:10.1186/s12872-022-02707-5)
Supplement: Supplementary file 3 — Additional file 3: Table 6. REACH-HF activity at individual Beacon Sites between June 2019 and June 2020. [file 12872_2022_2707_MOESM3_ESM.docx]

**Additional file 3**

**Table 6. REACH-HF activity at individual Beacon Sites between June 2019 and June 2020**

| Site | | Site 1 | Site 2 | Site 3 | Site 4 |
| --- | --- | --- | --- | --- | --- |
| Started treatment n (%) | | 61 (46.2%) | 35 (26.5%) | 26 (19.7%) | 10 (7.6%) |
| Completed treatment n (%) | | 42 (68.9%) | 28 (80%) | 23 (88.5%) | 3 (30%) |
| Average sessions received mean (SD) | | 9.2 (4.5) | 6.3 (2.9) | 6 (1.5) | 4.7 (2.8) |
| Treatment duration in days mean (SD) | | 144.7 (53.4) | 111.6 (26.7) | 96.6 (24.1) | 196 (64) |
| Dropped out n (%) | | 19 (31.1%) | 7 (20%) | 3 (11.5%) | 7 (70%) |
| Reason for not completing n (%) | DNA unknown reason | 1 (6.2%) | 5 (83.3%) | - | - |
|  | Left the area | 1 (6.2%) | - | - | - |
|  | Planned/emergency intervention | - | - | 1 (33.3%) | - |
|  | Too ill | 5 (31.2%) | 1 (16.7%) | 1 (33.3%) | - |
|  | Died | 3 (18.7%) | - | - | - |
|  | Hospital readmission | 2 (12.5%) | - | - | - |
|  | Other | 4 (25%) | - | 1 (33.3%) | 3 (100%) |
|  | Missing | 3 (15.8%) | 1 (14.3%) | - | 4 (57.1%) |

REACH-HF = Rehabilitation EnAblement in CHronic Heart Failure; SD = standard deviation; DNA = did not attend
