# Supplementary material for: A pragmatic effectiveness-implementation study comparing trial evidence with routinely collected outcome data for patients receiving the REACH-HF home-based cardiac rehabilitation programme
Source: BMC Cardiovasc Disord. 2022 Jun 16;22:270. doi: 10.1186/s12872-022-02707-5 (PMC9202968; doi:10.1186/s12872-022-02707-5)
Supplement: Supplementary file 4 — Additional file 4: Table 7a. Socio-demographic characteristics of patients enrolled onto the REACH-HF programme at individual Beacon Sites between June 2019 and June 2020 (including missing data) AND Table 7b. Comparison of patients receiving the REACH-HF intervention at the Beacon Sites between June 2019 and June 2020, those recruited into the REACH-HF trial and the general heart failure population recorded in the NACR database between June 2019 and June 2020 (excluding Beacon Sites patients). [file 12872_2022_2707_MOESM4_ESM.docx]

**Additional file 4**

**Table 7a. Socio-demographic characteristics of patients enrolled onto the REACH-HF programme at individual Beacon Sites between June 2019 and June 2020 (including missing data)**

| Site (n) | | Site 1 (61) | Site 2 (35) | Site 3 (26) | Site 4 (10) | Total (132) |
| --- | --- | --- | --- | --- | --- | --- |
| Mean age in years (SD) | | 80.5 (8.6) | 73.1 (10) | 71.3 (12.4) | 65.6 (12.1) | 75.6 (11.1) |
| Gender n (%) | Male | 39 (63.9%) | 27 (81.8%) | 13 (50%) | 7 (70%) | 86 (66.2%) |
|  | Not specified | - | 2 (5.7%) | - | - | 2 (1.5%) |
| Ethnic group n (%) | White | 52 (100%) | 5 (100%) | 15 (62.5%) | 9 (100%) | 81 (90%) |
|  | Non-white | - | - | 9 (37.5%) | - | 9 (10%) |
|  | Missing | 9 (14.7%) | 30 (85.7%) | 2 (7.7%) | 1 (10%) | 42 (31.8%) |
| Marital Status n (%) | Single | 16 (34.1%) | - | 11 (52.4%) | 2 (30%) | 29 (35.8%) |
|  | Married/living with a partner | 31 (65.9%) | 3 (100%) | 10 (47.6%) | 8 (70%) | 52 (64.2%) |
|  | Missing | 14 (22.9%) | 32 (91.4%) | 5 (19.2%) | - | 51 (38.6%) |
| Employment status n (%) | Employed | - | 2 (100%) | 2 (9.5%) | 1 (12.5%) | 5 (7.8%) |
|  | Unemployed/retired | 33 (100%) | - | 19 (90.5%) | 7 (87.5%) | 59 (92.2%) |
|  | Missing | 28 (45.9%) | 33 (94.3%) | 5 (19.2%) | 2 (20%) | 68 (51.5%) |
| Rural/urban indicator n (%) | Rural town and fringe | - | 12 (35.3%) | - | - | 12 (9.9%) |
|  | Rural village and dispersed | 1 (1.6%) | 8 (23.5%) | - | - | 9 (7.4%) |
|  | Urban city and town | 1 (1.6%) | 14 (41.2%) | - | - | 15 (12.4%) |
|  | Urban major conurbation | 59 (96.7%) | - | 26 (100%) | - | 85 (70.2%) |
|  | Missing | - | 1 (2.8%) | - | 10 (100%) | 11 (8.3%) |
| Index of multiple deprivation n (%) | Lowest quintile | 13 (21.3%) | 2 (5.9%) | 13 (50%) | - | 28 (23.1%) |
|  | Second quintile | 4 (6.5%) | 2 (5.9%) | 5 (19.2%) | - | 11 (9.1%) |
|  | Third quintile | 13 (21.3%) | 5 (14.7%) | 3 (11.5%) | - | 21 (17.4%) |
|  | Fourth quintile | 11 (18%) | 13 (38.2%) | 4 (15.4%) | - | 28 (23.1%) |
|  | Fifth quintile | 20 (32.8%) | 12 (35.3%) | 1 (3.8%) | - | 33 (27.3%) |
|  | Missing | - | 1 (2.8%) | - | 10 (100%) | 11 (8.3%) |
| Heart failure status at baseline n (%) | NYHA Class 1 | 2 (8.3%) | - | - | - | 2 (5.9%) |
|  | NYHA Class 2 | 20 (83.3%) | - | 2 (22.2%) | 1 (100%) | 23 (67.6%) |
|  | NYHA Class 3 | 2 (8.3%) | - | 7 (77.8%) | - | 9 (26.5%) |
|  | Missing | 37 (60.6%) | 35 (100%) | 17 (65.4%) | 9 (90%) | 98 (74.2%) |

REACH-HF = Rehabilitation EnAblement in CHronic Heart Failure; SD = standard deviation; NYHA = New York Heart Association Heart Failure Classification

**Table 7b. Comparison of patients receiving the REACH-HF intervention at the Beacon Sites between June 2019 and June 2020, those recruited into the REACH-HF trial and the general heart failure population recorded in the NACR database between June 2019 and June 2020 (excluding Beacon Sites patients)**

|  | **Beacon Sites (n=132, unless specified otherwise)** | **Trial data – treatment arm (n=96**, unless specified otherwise)** | **Heart failure patients recorded in the NACR for the same period (excluding Beacon Site patients) (n=5549)** | |
| --- | --- | --- | --- | --- |
| Rural/urban indicator – n (%) | Rural town and fringe 12 (9.9) |  |  | |
|  | Rural village and dispersed 9 (7.4) |  |  | |
|  | Urban city and town 15 (12.4) |  |  | |
|  | Urban major conurbation 85 (70.3) |  |  | |
| Index of multiple deprivation – n (%) | Lowest quintile 28 (23.1) |  |  | |
|  | Second quintile 11 (9.1) |  |  | |
|  | Third quintile 21 (17.4) |  |  | |
|  | Fourth quintile 28 (23.1) |  |  | |
|  | Fifth quintile 33 (27.3) |  |  | |
| Mean systolic blood pressure at baseline (SD, n) | 117 (15.8, 43) | 128.9 (19.2, 90) | 122.3 (20.1, 4299) | |
| Mean diastolic blood pressure at baseline (SD, n) | 65.1 (8.9, 43) | 71.1 (10.2, 90) | 71.7 (11.9, 4296) | |
| **Comorbidities – n (%)** | | | | |
| Angina | - | 18 (18.7) | 475 (10.9) | |
| Arthritis (osteo or rheumatoid) | 18 (13.6) | 41 (42.7) | 770 (17.7) | |
| Diabetes | 10 (7.6) | 25 (26) | 1304 (30) | |
| Stroke | 6 (4.5) | 11 (11.4) | 363 (8.3) | |
| Osteoporosis | 3 (2.3) | 6 (6.2) | 95 (2.2) | |
| Hypertension | 24 (18.2) | 40 (41.7) | 2038 (46.9) | |
| COPD (Chronic bronchitis or Emphysema) | 5 (3.8) | 9 (9.4) | Chronic bronchitis | 267 (6.1) |
|  |  |  | Emphysema | 196 (4.5) |
| Asthma | 4 (3) | 9 (9.4) | 421 (9.7) | |
| Chronic Back Problems | 16 (12.1) | 28 (29.2) | 467 (10.7) | |
| Depression | 7 (5.3) | 21 (21.9) | 380 (8.7) | |
| **Key disease-modifying medicines – n (%)** | | | | |
| Angiotensin-converting enzyme (ACE) inhibitors | 38 (28.8) | 62 (64.6) | 2223 (56) | |
| Angiotensin receptor blockers | 21 (15.9) | 26 (27.1) | 918 (23.1) | |
| Beta blockers | 72 (54.5) | 82 (85.4) | 3404 (85.8) | |
| Anticoagulant | 42 (31.8) | 42 (43.8) | 1383 (34.8) | |
| Aldosterone receptor antagonist | 23 (17.4) | 52 (54.2) | 1806 (45.5) | |

* Valid percent values; ** Baseline characteristics for 96 patients who had MLHFQ recorded at baseline and four-month follow-up, unless specified otherwise

REACH-HF = Rehabilitation EnAblement in CHronic Heart Failure; NACR = National Audit of Cardiac Rehabilitation; SD = standard deviation; COPD = chronic obstructive pulmonary disease
