# Supplementary material for: A pragmatic effectiveness-implementation study comparing trial evidence with routinely collected outcome data for patients receiving the REACH-HF home-based cardiac rehabilitation programme
Source: BMC Cardiovasc Disord. 2022 Jun 16;22:270. doi: 10.1186/s12872-022-02707-5 (PMC9202968; doi:10.1186/s12872-022-02707-5)
Supplement: Supplementary file 5 — Additional file 5: Table 8. Referral sources for patients enrolled on the REACH-HF programme at individual Beacon Sites between June 2019 and June 2020. [file 12872_2022_2707_MOESM5_ESM.docx]

**Additional file 5**

**Table 8. Referral sources for patients enrolled on the REACH-HF programme at individual Beacon Sites between June 2019 and June 2020**

| Site (n) | | Site 1 (60) | Site 2 (32) | Site 3 (12) | Site 4 (1) |
| --- | --- | --- | --- | --- | --- |
| Source of referral n (%) | Consultant | 3 (5%) | - | 2 (16.7%) | - |
|  | Cardiac nurse | 55 (91.7%) | 32 (100%) | 8 (66.7%) | 1 (100%) |
|  | GP | 1 (1.7%) | - | - | - |
|  | Primary care nurse | 1 (1.7%) | - | 2 (16.7%) | - |
|  | Missing | 1 (1.6%) | 3 (8.6%) | 14 (53.8%) | 9 (90%) |

REACH-HF = Rehabilitation EnAblement in CHronic Heart Failure
