# Supplementary material for: A pragmatic effectiveness-implementation study comparing trial evidence with routinely collected outcome data for patients receiving the REACH-HF home-based cardiac rehabilitation programme
Source: BMC Cardiovasc Disord. 2022 Jun 16;22:270. doi: 10.1186/s12872-022-02707-5 (PMC9202968; doi:10.1186/s12872-022-02707-5)
Supplement: Supplementary file 6 — Additional file 6: Table 9. Effect sizes for primary and secondary outcome measures at individual Beacon Sites for patients receiving REACH-HF programme between June 2019 and June 2020. [file 12872_2022_2707_MOESM6_ESM.docx]

**Additional file 6**

**Table 9. Effect sizes for primary and secondary outcome measures at individual Beacon Sites for patients receiving REACH-HF programme between June 2019 and June 2020**

|  | | Site | Pre-treatment | | | Post-treatment | | | Effect size  Hedges’ *g*  (95% CI) |
| --- | --- | --- | --- | --- | --- | --- | --- | --- | --- |
|  |  |  | n | Mean | SD | n | Mean | SD |  |
| HRQoL | MLHFQ | S1 | 7 | 15.9 | 19.7 | 7 | 18.9 | 18.7 | 0.15  (-0.90, 1.20) |
|  |  | S2 | 19 | 31.2 | 21.9 | 19 | 30.5 | 23.5 | -0.03  (-0.67, 0.61) |
|  |  | S3 | 22 | 43.3 | 18 | 22 | 38.6 | 16.7 | -0.27  (-0.86, 0.33) |
|  |  | S4 | 2 | 75 | 5.7 | 2 | 70 | 14.1 | -0.27  (-2.74, 2.20) |
|  | COOP | S1 | 3 | 17.4 | 7 | 3 | 21 | 7 | 0.41  (-1.24, 2.06) |
|  |  | S2 | - | - | - | - | - | - | - |
|  |  | S3 | 1 | 21.9 | 14 | 1 | 18.7 | 14 | Not estimable |
|  |  | S4 | - | - | - | - | - | - | - |
| Mental health | HADS (depression) | S1 | 7 | 2.9 | 3.1 | 7 | 5.1 | 4.7 | 0.52 (-0.55, 1.59) |
|  |  | S2 | - | - | - | - | - | - | - |
|  |  | S3 | 14 | 6.9 | 4.4 | 14 | 5.7 | 4.2 | -0.27 (-1.02, 0.47) |
|  |  | S4 | 2 | 15 | 2.8 | 2 | 8 | 2.8 | -1.43 (-9.75, 6.89) |
|  | PHQ-9 | S1 | - | - | - | - | - | - | - |
|  |  | S2 | 17 | 7.4 | 5.6 | 17 | 6.2 | 4.7 | -0.23 (-0.90, 0.45) |
|  |  | S3 | - | - | - | - | - | - | - |
|  |  | S4 | - | - | - | - | - | - | - |
|  | HADS (anxiety) | S1 | 7 | 4 | 4.8 | 7 | 4.7 | 4 | 0.15 (-0.90, 1.20) |
|  |  | S2 | - | - | - | - | - | - | - |
|  |  | S3 | 14 | 7.4 | 3.8 | 14 | 6.3 | 3.8 | -0.28 (-1.03, 0.46) |
|  |  | S4 | 2 | 17 | 2.8 | 2 | 14 | 1 | -0.82 (-5.83, 4.20) |
|  | GAD-7 | S1 | - | - | - | - | - | - | - |
|  |  | S2 | 17 | 4.1 | 3.3 | 17 | 3.3 | 3.3 | -0.24 (-0.91, 0.44) |
|  |  | S3 | - | - | - | - | - | - | - |
|  |  | S4 | - | - | - | - | - | - | - |
| Objective exercise capacity assessment | ISWT (m) | S1 | 2 | 160 | 56.6 | 2 | 135 | 7.1 | -0.35  (-3.16, 2.45) |
|  |  | S2 | - | - | - | - | - | - | - |
|  |  | S3 | 10 | 196 | 135.7 | 10 | 229 | 135.8 | 0.23  (-0.65, 1.11) |
|  |  | S4 | 1 | 190 | - | 1 | 190 | - | Not estimable |
|  | 6MWT (m) | S1 | - | - | - | - | - | - | - |
|  |  | S2 | - | - | - | - | - | - | - |
|  |  | S3 | 1 | 83 | - | 1 | 166 | - | Not estimable |
|  |  | S4 | 1 | 190 | - | 1 | 280 | - | Not estimable |

REACH-HF = Rehabilitation EnAblement in CHronic Heart Failure; SD = standard deviation; CI = confidence intervals; MLHFQ = Minnesota Living with Heart Failure Questionnaire; COOP = Dartmouth Cooperative Functional Assessment Charts; HADS = Hospital Anxiety and Depression Scale; PHQ-9 = Patient Health Questionaire-9; GAD-7 = Generalised Anxiety Disorder Assessment-7; ISWT = Incremental Shuttle Walk Test; 6MWT = Six Minute Walk Test
